# Testing the Accuracy of Different A-Axis Types for Measuring the Orientation of Bones in the Archaeological and Paleontological Record

**DOI:** 10.1371/journal.pone.0068955

**Published:** 2013-07-12

**Authors:** Manuel Domínguez-Rodrigo, Alfonso García-Pérez

**Affiliations:** 1 Instituto de Evolución en África (IDEA), Museo de los Orígenes, Madrid, Spain; 2 Department of Prehistory, Complutense University, Madrid, Spain; 3 Department of Statistics, Universidad Nacional de Educación a Distancia (UNED), Madrid, Spain; University of Oxford, United Kingdom

## Abstract

Orientation of archaeological and paleontological materials plays a prominent role in the interpretation of site formation processes. Allochthony and authochthony are frequently assumed from orientation patterns or lack thereof. Although it is still debated to what extent orientation of items can be produced in original depositional contexts, the recent use of GIS tools to measure orientations has highlighted several ways of reproducing A-axes with which to address these taphonomic issues. In the present study, the three most relevant A-axis types are compared to test their accuracy in reproducing water current direction. Although results may be similar in specific bone shapes, differences are important in other shapes. As known in engineering working with wind and fluid mechanics (developing shape optimization), longitudinal symmetrical axes (LSA) are the one that best orient structures against or in the same direction of wind and water. The present work shows that this is also the case for bones (regardless of shape), since LSA produce the most accurate estimates of flow direction. This has important consequences for the interpretation of orientation patterns at sites, since this type of axis is still not properly reproduced by GIS available tools.

## Introduction

Taphonomic research has revealed a plethora of processes that can potentially modify archaeological and paleontological records from their original depositional states. These processes introduce bias in the understanding of site formation history and in the resulting behavioural and ecological interpretations of such modified assemblages. Orientation patterns in fossil and stone tool assemblages are among the key indicators that paleontologists and archaeologists use to interpret the degree of site disturbance. Assemblages affected by water disturbance may adopt a variety of forms, ranging from (peri)autochthonous rearranged assemblages and biased lag assemblages to transported assemblages. A substantial amount of research has been aimed at evaluating the degree of distortion introduced by physical processes (namely, water flows and, to a lesser extent, slope gravity) in paleontological and archaeological sites during the biostratinomic stage of their formation (see reviews in [1], [2], [3]). The diverse variables most frequently used to infer physical disturbance include the following: sedimentology, where mineral grain size selection is suggestive of depositional energy; preferential orientation of items [4], [5], [6]; the presence of physical signs of modification caused by transportation, such as rounding, polishing and abrasion [7], [8]; for bones, differential anatomical representation according to element type [6], [9]; and for lithic assemblages, a combination of quantitative variables based on average weight, a large-to-small artefact ratio and the relative representation of the fraction <50 g, where those assemblages affected by water show high values for the former two variables and low values for the latter [1].

Several of these variables are equivocal and lend themselves to equifinality. Among these variables, item orientation is still the most widely used variable to infer sorting or rearrangement of archaeological and paleontological materials due to hydraulic processes. Experiments have shown that long axes of bones and, to a lesser extent, stone tools react to current direction and force by aligning: parallel (preferentially) to the water flow when completely covered by water; more often transversal to it when in shallow water or partially exposed to the surface; or forming criss-cross patterns [4]–[8], [10]. However, the possibility that orientation could also have other causes has never been discarded. From a taphonomic point of view, this is important vis-a-vis producing reliable criteria to differentiate processes generating orientation patterns that could erroneously be interpreted as the resulting action of water winnowing by rivers or lakes. For example, trampling has been shown to produce movement of bones and artefacts, predominantly in the same direction as the moving trampling agent, and sometimes transversal to it [11], [12]. Downslope gravity has also been argued to create differential movement of bones and re-oriented assemblages [9], [13]–[15].

Orientation patterns of fossil assemblages have been used to infer various degrees of autochthony or allochthony [2], [3]. To orient objects, several protocols have been used. A widespread protocol among taphonomists is the use of A-axes (maximum length axes) that are at least twice as long as B-axes (maximum breadth axes), taking as A-axis the line that divides the object approximately symmetrically [3], [16]. This is referred to as a Symmetrical Longitudinal Axis (SLA) [3], [17]. Symmetrical longitudinal axes play a major role in the orientation of objects in physics and engineering, both in aerial (wind) and fluid (e.g., water) environments, in which objects only project themselves straight against currents or move along with them properly when symmetrical properties along a longitudinal axis are met (e.g. [18]–[26]). SLA is commonly used in paleontological research on site formation processes, in which bone orientation is taken parallel to the vertical plane that includes the long axis of the bone [2], [4], [16]. However, it should be stressed that, most commonly, the definition of the A-axis is self understood and therefore, taphonomic work does not describe in detail how A-axes were established for every shape of measured bone.

**Figure 1:**
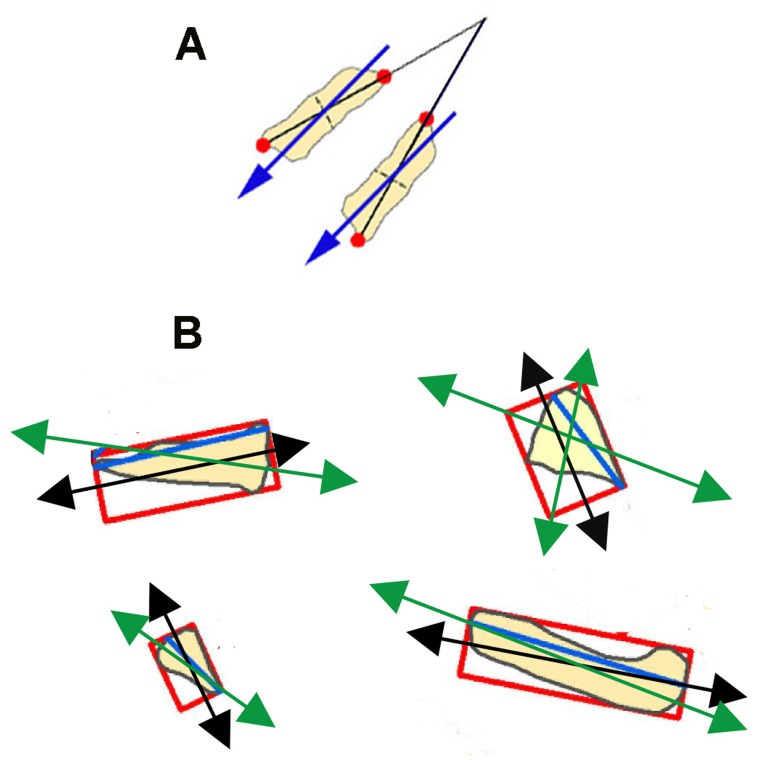
Comparison of different A-axis types. A, Contrast in orientation between DPA [1] and SLA (blue arrows). Notice how two parallel specimens with identical longitudinal axes (SLA) show divergent DPA of 30°. B, Some examples from [14] showing MBRA (black arrows), DPA (blue lines) and SLA (green arrows). Notice the divergent angles.

The recent introduction of GIS tools that automatically take the orientations of objects on rasterized or digitized plans has modified the SLA protocol by introducing alternative types of A-axes. One of these alternative A-axes is the Diameter Polygon Axis (DPA) [2], [27], which consists of taking the maximum length of the object, regardless of whether it divides the object symmetrically or not (Figure 1). A GIS proxy for SLA has been reproduced by using the Minimum Bounding Rectangle Axis (MBRA), which consists of encasing each object with a rectangle and taking the symmetrical axis of the rectangle, assuming that it reproduces the symmetrical axis of the object [27]. Although important differences between MBRA and SLA exist (see Figure 1) one could theoretically assume that such differences are not significant at the assemblage level. However, DPA has been argued to be suitable to estimate the elongation direction of irregularly shaped items [27]. Both assumptions about MBRA and DPA are inferentially derived and remain untested experimentally. Although DPA has been used extensively to infer orientation patterns from the Olduvai Gorge sites [2], [27], the overwhelming majority of the fossil objects from these sites are elongated; therefore DPA (theoretically, more apt for irregularly-shaped objects) may be biasing the inferences made from the results. Given that most objects in paleontological and archaeological assemblages are elongated, it is important to know which type of A-axis better detects the true orientation of objects in any given assemblage. It is also important to know what role the object shape plays in orientation and how this orientation is best reproduced when using one or another type of A-axis.

Recent experimental work shows that bone shape and texture play an important role in bone transport [17]. Tubular long bones and polygonal compact bones are preferentially transported over flat bones. Among the latter, flat bones with a trabecular texture are also preferentially transported over flat dense bones resulting from the breaking of long bones [17]. This is documented regardless of bone size. Although in such an experimental study, SLA produced better estimates of the direction of the water current than DPA, it was observed that part of this was caused because the most outlying values for DPA occurred in irregularly-shaped bones. Therefore, new experiments were required to address the effect that shape plays in object orientation with respect to a fixed current direction. With the exception of compact bones and vertebrae, which are easily transported away from any assemblage by low-energy water currents [6], most shape diversity in bones (especially irregular shapes) occur in (semi-) flat broken elements, such as mandibles, ribs, scapulae, pelves and fragmented long bone shafts. These are the bulk of archaeological assemblages. These (semi-) flat specimens can be divided into two different shapes: elongated (when the A-axis is >2 times the size of the B-axis), and irregular (when the A-axis is <2.0 and the contour of the specimen cannot fill most of a rectangular frame, because there are more than two similarly long sides). Irregular shapes can be triangular (such as scapulae) or polygonal (such as pelves and some fragmented long bone shafts). In theory, irregularly shaped objects would tend to find an axis of symmetry to stabilize against currents. However, how this symmetry axis is identified may differ from clearly elongated objects.

It has been acknowledged that there is not a single method for computing the shape orientation of all shapes [28]. The present work will focus on this topic and not on bone transport, to provide a referential framework to understand which types of axis should be used when taking orientation measurements from bones according to their shapes. The null hypotheses addressed here will be: 1) that no type of A-axis provides more accurate results than the others in measuring the trend, 2) that no link exists between shape and A-axis type, and 3) that there are no differences in orientation patterns and mean vectors according to elongation index.

## Methods

### Sample and Experiment Protocol

**Figure 2:**
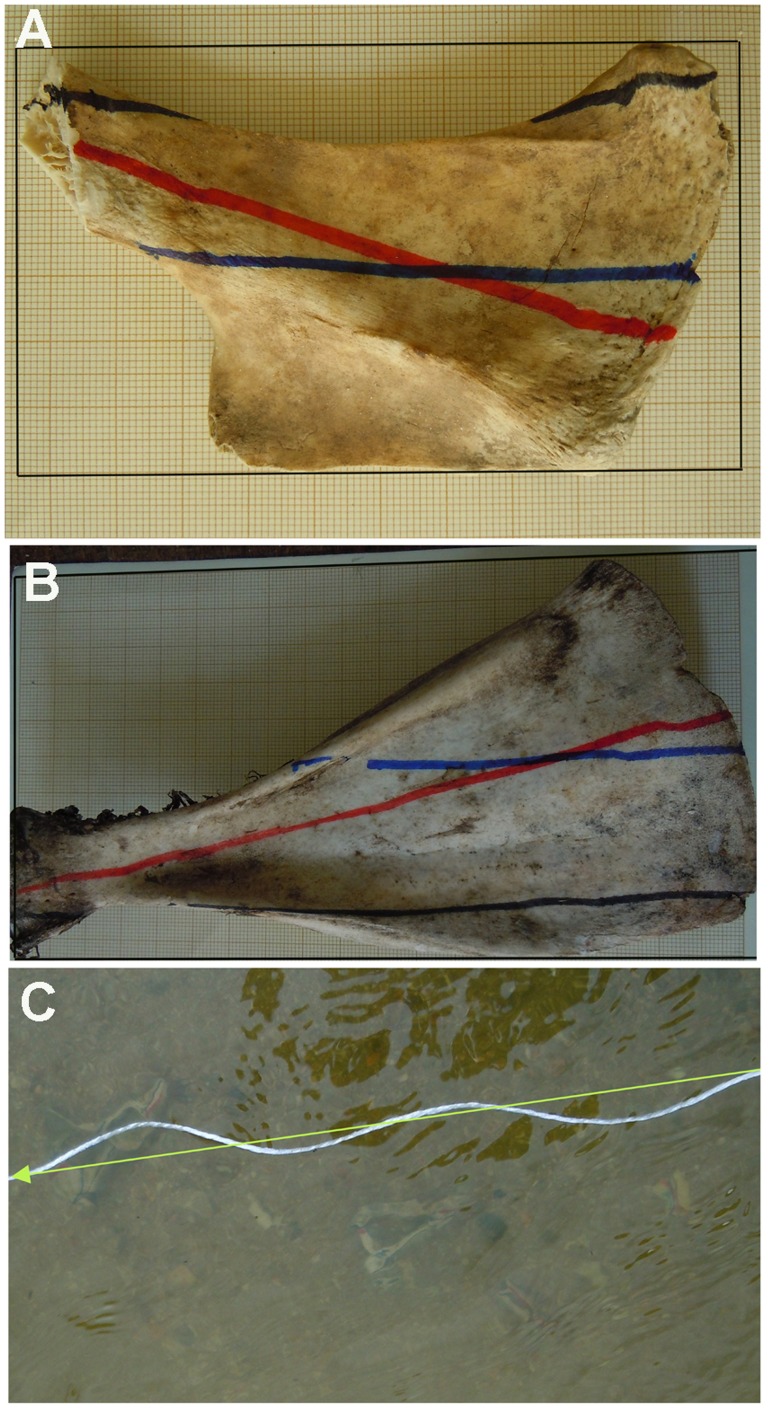
Orientation patterns of bones with different shapes. A and B, examples of SLA (red), DPA (black) and MBRA (blue) in irregular shaped bones. C, fluvial experiment with bones oriented and water current direction indicated by arrow and string.

A sample of 136 bone specimens from adult deer and pig, and a subadult cow were used in a fluvial experiment. The analysis was carried out on the retrieved sample of 82 bones. Bones were divided into three shapes: longitudinal tubular (30 long bones representing 2 humeri, 5 femora, 5 tibiae and 3 radii), longitudinal flat (19 long bone shafts plus 9 rib fragments) and irregular flat (13 pelves, 6 scapulae, 3 long bone shafts, 1 mandible, 1 thoracic vertebra). Irregular bones usually showed three or more long sides, instead of two as is typical of longitudinal bones. Three types of A-axes (SLA, DPA and MBRA) were drawn in different colours with permanent markers on the bones. SLA was taken as the maximum length of a symmetrical axis dividing the element in two similar sides. DPA was taken as the maximum length dimension. MBRA was created after setting each bone in a rectangle and then the symmetrical axis of the rectangle was drawn (Figure 2). Bones were subsequently placed in the Guadarrama river in its course between Boadilla del Monte and Brunete in Madrid. Bones were randomly and jointly dropped along a 4 meter transect on a shallow portion of the river side near a point bar at a depth of 25 cm. Water velocity was measured with a paddle velocimeter at 68 cm per second. A string attached to a pole was placed 2 meters before the location where bones were dropped. A cork was attached to the end of the string, which then showed the direction of the current. The string axis was taken as the flow direction axis. Bones were exposed to the current for two consecutive hours. Afterwards, a compass was used to measure each of the three axes on each bone prior to collection. Each measurement took some time, because the strong current created some unstable surfaces and this may have caused some refraction. To overcome this, measurements were taken repeatedly until no more than a two degree difference was obtained. Posteriorly we introduced a control to evaluate the impact that refraction might have on measurements taken directly on the bone axes as perceived from the surface. The axes of a set of bones (n = 5) were reproduced on a surface by using an artificial double connected axis. This device (comprising two parallel straight axes separated by a 30 cm thin pole) was placed under water above each of the three types of axis of each bone and then measured by the orientation of the surface axis. Comparisons between the measurements of each axis from underwater bones as observed above surface and the superficial axis showed minimal distortion in all cases (<5 degrees) and when documented this distortion affected all axes equally. The minimal differences between both types of measurements can probably be explained by the fact that the experiment was conducted under the shade of a bridge, where the light was not reaching underwater at an angle. This showed that measurements taken from the observation of underwater axes could be confidently used with minimal distortion. Orientation data (in degrees) were added to a database, which included each bone type, length, breadth, elongation index, structure (dense or trabecular) and element and section type. Graphic representation of angular axial histograms was made with Oriana 4.0.

Orientation analyses were carried out using the complete sample and then using subsamples according to bone shape. Subsequently, orientation analyses were made on three subsamples using a different elongation index for each: short (from 1.2 to 1.4), intermediate (from 1.4 to 1.8) and long (>2.0). In the present experiment no bone provided an elongation index between 1.8 and 2.0. The elongation index was obtained by dividing the maximum dimension of the A-axis (length) by the maximum dimension of the B-axis (breadth).

No permission was requested for the use of these faunal elements to any ethics committee because the bones were obtained from a commercial butcher (Ben-Car, Boadilla del Monte, Madrid), except for the deer bones, which were collected at the Hosquillo reserve (Cuenca, Spain). Hosquillo Reserve granted permission for research. Some of these materials were previously used in other experimental research [17]. The study was carried out on public land and no permission was necessary given its non-contaminant or non-environmentally modifying nature. This experiment did not involve any endangered or protected species.

### Statistical Analysis

Isotropy, uniformity (or randomness in orientation) is of great importance in directional data analysis. To test whether a circular distribution of data is random (null hypothesis) or non-random (alternative), non-parametric omnibus tests like Kuiper’s (*V*) test and Watson’s (*U^2^*) test [29] are recommended. Traditional linear tests, such as the Kolmogorov-Smirnov test, are not appropriate because the values of this test depend on the choice of the origin in the directional data. For this reason, this test is not suitable for this kind of data. Precisely, the invariant versions of such test, appropriate for circular data, are Kuiper’s and Watson’s tests. Furthermore, it has been shown that Kuiper’s test is more efficient than Kolmogorov-Smirnov test in the sense of Bahadur efficiency in situations where both are applicable as on the real line.

It is also possible to test uniformity against specific parametric models using Rayleigh’s (*R*) test. A model for assessing the normal distribution of circular data is the von Mises distribution. For this distribution, the dispersion is quantified by a concentration parameter *k*, where *k* = 0 corresponds to an isotropic distribution and increasing values reflect a trend towards anisotropy.

Values with p>0.05 indicate that the null hypothesis of isotropy cannot be rejected. The three tests were applied in the present study. A V-test of the null hypothesis of isotropy against the alternative direction of the water current was also applied [29]. These tests were carried out with Oriana 4.0.

To avoid circumstantial results owed to the stochastic nature of the diversity of specimen sizes and shapes, which could be greatly influenced by outliers, a robust approach was undertaken for the analysis of the circular data produced by the experiment. Data in degrees were transformed to radians prior to their analysis.

Directional data are those in which measurements are directions. This is the case of the data considered in this paper. If traditional (linear) methods are used for this kind of data (e.g., the sample mean), we can obtain meaningless estimations (e.g., the linear sample mean of directions 20° and 340° would be (20+340)/2 = 180°, pointing at the wrong direction, instead of the suitable 360° or 0°). For this reason, we also estimated the items’ orientation and their relationship to the current direction with the sample circular mean direction.

Because data may contain outliers, it is convenient accompany classical estimations with robust ones. If 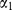, …, 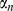
are a set of circular observations given in terms of angles and we obtain the resultant vector of these n unit vectors by adding them component-wise, to get
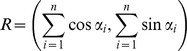
the direction of this resultant vector *R* is the sample circular mean direction that is denoted by 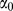
as the classical estimator of the circular mean direction θ (See [30]). The computation of this can be done with the function “circ.mean” of the R library “CircStats”, or equivalently by the function “mean.circular” of the R library “circular” [31].

Since outliers can appear within the observations, it is better to compute robust circular estimates. Because circular variables are bounded, we have a problem even to define what is an outlier for circular data. We consider that outliers are observations that are highly unlikely to occur under the assumed model *f*. In this work we will assume a Von Mises model as underlying model and we will consider a minimum disparity measure (MDE) estimator 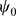 as a robust estimator that minimizes the Hellinger distance between the density estimator 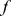 and the underlying model 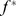:
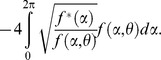



If we represent by
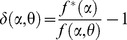
and ω(α;θ, 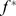) the positive part of the following quantity



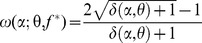
the MDE estimator 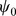 is the solution in θ of the estimating equation



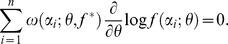
(See [32] for details).

The computation of this robust estimator is done with the function “mde.vonmises” of the R library “wle” [31].

## Results

**Figure 3:**
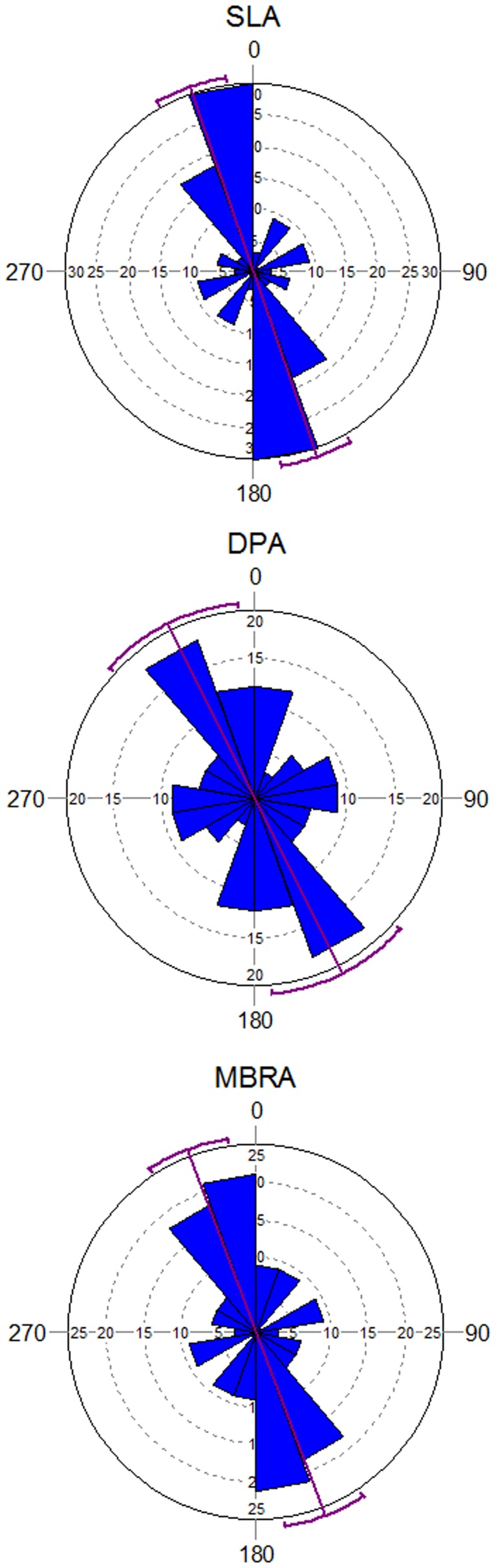
Rose diagrams of the complete bone assemblage with histograms of preferred orientations, according to A-axis type (SLA; DPA, and MBRA). Mean vectors (purple line) and confidence intervals (arcs) are shown. Reliable intervals appear in the same colour as the mean vector. Unreliable intervals are shown in red.

A total of 82 bone specimens stayed at the depositional spot, while 54 specimens were transported away by the strong water current. Most of the transported bones were trabecular in structure (rib and pelvis fragments), confirming previous analyses of the preferential transportability of these types of elements by water currents [3], [6]. Analysis of the autochthonous assemblage yielded anisotropy (Table 1). Elongated bones aligned (preferentially) parallel to the water current and, to a lesser extent, transversally to it (Figure 3). Rayleigh’s, Kuiper’s and Watson’s tests significantly support (p<0.05) that the assemblage showed a strong preferred orientation regardless of the A-axis type. When considering each type of axis, SLA showed a mean vector closer to the direction of the water current, with a higher concentration value and smaller circular variance than the other two axis types. This reflects that this axis type indicates the current direction more accurately than the other two axis types.

**Table 1:** Concentration values, mean direction, basic statistics and omnibus tests of the three axis types.

A-axis type	SLA	DPA	MBRA
Mean Vector (µ)	161.016	153.446	159.21
Length of Mean Vector (r)	0.393	0.201	0.346
Concentration (k)	0.856	0.411	0.737
Circular Variance	0.303	0.399	0.327
Circular Standard Deviation	39.128	51.286	41.745
95% Confidence Interval(−/+) for µ	150.262	131.763	146.852
	171.77	175.129	171.568
One Sample Tests			
Rayleigh Test (Z)	12.54	3.286	9.69
Rayleigh Test (p)	0.0000	0.037	0.000
Watson’s U^2^ Test (Uniform, U^2^)	0.892	0.289	0.633
Watson’s U^2^ Test (p)	<0.005	<0.01	<0.005
Kuiper’s Test (Uniform, V)	3.656	2.397	3.146
Kuiper’s Test (p)	<0.01	<0.01	<0.01
V Test (V; expected mean 170.00)	0.389	0.193	0.34
V Test (u)	4.947	2.457	4.324
V Test (p)	0.000	0.007	0.000

When considering the complete assemblage, regardless of individual bone shape, both SLA (3.08) and MBRA (3.07) provide the closest estimates to the mean direction of the river current at the section of the channel where the experiment was carried out (170°; rad = 2.96) (Table 2). DPA (3.15) shows a significant distance from the other two axes and the mean current direction. This is also documented when the assemblage is divided in sub-assemblages according to bone shape. In longitudinal flat bones (long bone shaft and rib fragments), SLA (3.81) and MBRA (3.81) provide a much closer estimate to the mean direction of the current than DPA (3.90). This is not documented in longitudinal tubular bones (long bone shafts plus ends), where the three axes are very similar (Table 2), but is observed again in irregular bones, where MBRA (3.04) and SLA (3.06) offer the closest estimate to the current direction of all the bone shapes, in contrast with DPA (3.12).

**Table 2:** Mean vector of each axis type, according to classical circular statistics and robust statistics.

	SLA		DPA		MBRA	
	mean	mde-v.Misses	mean	mde-v.Misses	mean	mde-v.Misses
all shapes	3.084984	3.064	3.151651	3.123185	3.072541	3.05
longitudinal flat	3.818045	3.900185	3.963162	4.000185	3.818045	3.900185
longitudinal tubular	2.673848	2.665	2.669438	2.685	2.663077	2.657
irregular flat	3.066266	3.058	3.129173	3.144	3.041211	3.025

**Figure 4:**
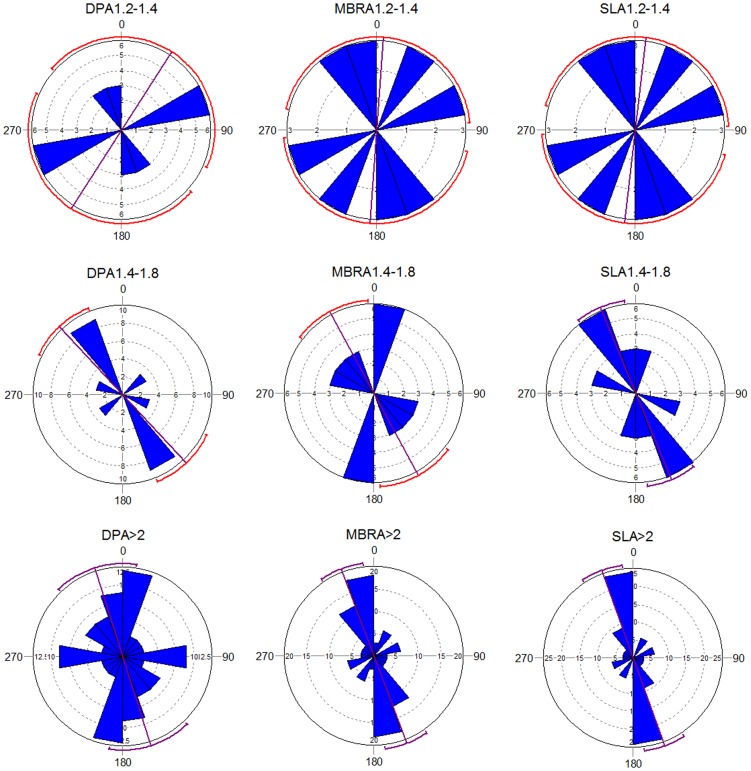
Rose diagrams of the sub-assemblages created by elongation index type (short, intermediate and long) with histograms of preferred orientations, according to A-axis type (SLA; DPA, and MBRA). Mean vectors (purple line) and confidence intervals (arcs) are shown. Reliable intervals appear in the same colour as the mean vector. Unreliable intervals are shown in red.

How does bone elongation affect orientation? Interestingly, specimens showing a short elongation index (n = 12) fail to provide evidence of anisotropy (Table 3; Figure 4). Rayleigh’s, Kuiper’s and Watson’s tests support isotropy. Furthermore, the mean vector of this sub-assemblage is extremely distant from the water current orientation. Intermediately elongated bones (n = 15) display unimodal anisotropy depending on the type of A-axis, and multimodal regardless of axis type (Table 3). In contrast with bones with a short elongation index, intermediate elongation shows mean vectors closer to the water current direction, but they are inferior to mean vectors produced by long elongation bones (n = 55), as also shown by the omnibus tests. *Z, V* and *U^2^* values for Rayleigh’s, Kuiper’s and Watson’s tests are between 35% (SLA) and 65% (MBRA) lower in intermediate elongation index than for the same axes in long elongation index (Table 3). Once again, DPA is highly fluctuant regardless of elongation index, suggesting its unreliability as orientation indicator. A long elongation index shows a mean vector with a difference of 11° (MBRA) and less than 10° (DPA, SLA) with respect to the water current direction, whereas an intermediate elongation index, the difference of the mean vector and the water current direction ranges between 33° (DPA) and 13° (SLA). Figure 4 shows that an intermediate elongation index using DPA and MBRA produce different trends from those resulting from the use of the same axes on a sub-assemblage of bones with a long elongation index. The highest similarity in trends when comparing the intermediate elongation index and the long elongation index can be observed on the SLA. Furthermore, both DPA and MBRA produced unreliable confidence intervals in the intermediate elongation sub-assemblage, as indicated in Figure 4 by the red arc displaying the confidence interval. In contrast, both the intermediate and the long elongation index produced reliable confidence estimates when using SLA. It could be argued that sample size is responsible for the lack of any detectable anisotropic pattern in the bone sub-assemblage showing a short elongation index. However, the sample of bones with intermediate elongation indices is similarly small and, in contrast, these produce clear anisotropic patterns that are similar to those reproduced by bones with a long elongation index (Figure 4). This was independently documented in experiments using a larger sample of bones with these three index types, where it was reported that the smallest specimens and those displaying a short elongation index showed more variable orientation directions [17]. These results caution against using items with a long elongation index to measure orientation with accuracy.

**Table 3:** Circular statistics for each of the three A-axis types according to elongation index: from 1.2 to 1.4, from 1.4 to 1.8 and >than 2.0.

Axis type	SLA1.2–1.4	DPA1.2–1.4	MBRA1.2–1.4	SLA1.4–1.8	DPA1.4–1.8	MBRA1.4–1.8	SLA>2	DPA>2	MBRA>2
Mean Vector (µ)	6.418	32.5	4.274	157.4	136.987	151.708	160.046	162.442	159.384
Length of Mean Vector (r)	0.2	0.044	0.168	0.604	0.477	0.431	0.396	0.205	0.379
Concentration	0.002	0	0	1.437	0.96	0.816	0.862	0.42	0.819
Circular Variance	0.4	0.478	0.416	0.198	0.261	0.284	0.302	0.397	0.31
Circular Standard Deviation	51.363	71.714	54.076	28.773	34.856	37.148	39	50.965	39.903
95% Confidence Interval (−/+) for µ	*****	*****	*****	141.837	115.56	127.268	146.962	136.424	145.669
	*****	*****	*****	172.964	158.414	176.148	173.129	188.459	173.099
One Sample Tests									
Rayleigh Test (Z)	0.482	0.023	0.34	5.47	3.413	2.792	8.463	2.28	7.759
Rayleigh Test (p)	0.627	0.978	0.72	**0.003**	**0.03**	0.059	**0.000211**	**0.102**	**0.000427**
Watson’s U^2^ Test (Uniform, U^2^)	0.125	0.134	0.113	0.379	0.349	0.234	0.638	0.226	0.538
Watson’s U^2^ Test (p)	0.25>p>0.15	0.15>p>0.1	0.25>p>0.15	<0.005	<0.005	<0.025	<0.005	<0.025	<0.005
Kuiper’s Test (Uniform, V)	1.639	1.537	1.557	2.477	2.363	2.159	3.14	2.093	2.931
Kuipers Test (p)	0.10>p>0.05	>0.15	0.15>p>0.10	<0.01	<0.01	<0.01	<0.01	<0.01	<0.01
V Test (V; expected mean 170.00)	−0.192	−0.032	−0.163	0.589	0.4	0.41	0.39	0.204	0.373
V Test (u)	−0.942	−0.157	−0.799	3.228	2.191	2.243	4.052	2.117	3.872
V Test (p)	0.824	0.562	0.785	0.000405	0.014	0.012	0.0000189	0.017	0.0000425

***** indicates that a result could not be calculated.

In sum, the null hypothesis 1 (no type of A-axis provides more accurate results than the others in measuring the trend) has been rejected by the present study, which shows that DPA is a deficient axis type to reproduce azimuth trends. Null hypothesis 2 (no link exists between shape and A-axis type) is supported by the present study, since in all the bone shapes, SLA and MBRA were the optimal A-axis types to reproduce the water current direction. Null hypothesis 3 (there is no differences in orientation patterns and mean vectors according to elongation index) has also been rejected by the present study which shows that a short elongation index fails to detect anisotropy, when an intermediate index and a long elongation index statistically show it, and that a long elongation index reproduces more faithfully the anisotropy and the mean vector indicating the water current direction. These results reinforce the use of the traditional taphonomic approach of measuring bone orientation using a symmetry A-axis and using a long elongation index [6], [16].

## Discussion

Experimental work with heavy-duty stone tools (i.e., handaxes) has shown that there are variable dispersions of orientations of these objects when they are exposed to currents and, therefore, several measurements are required to obtain accurate results [33]. However, this is probably due to the small number of experimental items in that study (n = 5), whose dimensional properties are heterogeneous and therefore may react differently to the same hydraulic flows. Most importantly, interpretations derived from the study of lithics do not apply to bones, since it was experimentally shown that bones react more efficiently to water flow than lithics and tend to show very early anisotropic patterns [8], [17]. A recent experiment with bones, including hundreds of measurements, has shown that very small sample sizes reproduce the anisotropic model of the population from which they are derived [17]. The population was repeatedly sampled by randomly selecting 100, 50 and 25 specimens and testing if they reproduced the anisotropic pattern of the assemblage. Omnibus tests showed that these subsamples reproduced the anisotropy of the population even when sample size is as low as 25 [17].

The sample size for the three bone shapes used in the present study is therefore within the range of reliability for inferring anisotropy, as demonstrated by experiments based on different sample sizes [17]. Results of the present study have shown that SLA and MBRA are very similar indicators of current directionality, probably because the latter is a good proxy of the former. Despite this, MBRA shows a smaller concentration value and a higher variance and standard deviation probably because, whereas in elongated specimens, this axis type faithfully reproduces a symmetrical axis, in irregular bones, it frequently may not (see Figure 2). Both types of A-axis are much better indicators of current directionality than DPA, and, therefore, are more suitable to infer proper anisotropic patterns of bone assemblages. These results advise against the use of DPA for reconstructing isotropy/anisotropy in archaeological and paleontological assemblages [2], [3], especially when these are not exclusively constituted of elongated items. Contrary to previous experimentally unsupported assertions that DPA is suitable to estimate the elongation direction of irregularly shaped items [27], the present experiment shows that this is not the case. SLA and MBRA in irregularly-shaped bone specimens showed the closest mean orientation to the water current. direction (Table 2). Contrary to our expectations, the mean direction of SLA and MBRA in irregularly-shaped bones was closer to the water current direction than elongated specimens. This may be due to the fact that the smaller dimension of B-axes in elongated items facilitates their quicker stabilization, which does not have to be perfectly parallel to the current direction. In contrast, irregular specimens stabilize once the current does not encounter a strong unbalance in the area of resistance to the current, which the specimen avoids by moving its SLA as parallel to the water current as possible. This principle is also observable in aeronautics and structural physics [23], [26].

Although SLA and MBRA can be used interchangeably, the higher vector length values and the higher unimodal and multimodal values of the omnibus tests in SLA, together with its more reduced range in the 95% confidence interval of the mean vector and its closer value to the water current direction (Table 1), show that SLA displays an increased accuracy in determining the true trend of any anisotropic assemblage. This is of utmost relevance because SLA cannot so far be automatically reproduced by any GIS software, in contrast with MBRA. For this reason, the time-consuming process of measuring SLA cannot be currently replaced with any automatic approach if accuracy is targeted (e.g., 2,17, 27]. Alternative methods, such as the use of MATLAB [33], also do not guarantee that SLA can be automatically obtained in items that are not symmetrical.

SLA, taken as the vertical plane of the elongation axis [16], has been the most frequent axis used for measuring orientation and suggesting different data graphic representations (e.g., [4], [34], [35], [36]). The present study has provided a solid experimental protocol for the use of the correct type of A-axis when analyzing isotropy/anisotropy in bone assemblages and vindicates the use of SLA over other A-axis types, as has been traditionally done by taphonomists (e.g., [3], [9], [16], [17], [3], [4,15]).
