# Correction: Testing the Accuracy of Different A-Axis Types for Measuring the Orientation of Bones in the Archaeological and Paleontological Record

**DOI:** 10.1371/annotation/16f3a072-218b-4413-a6e8-72f072735f1e

**Published:** 2013-12-16

**Authors:** Manuel Domínguez-Rodrigo, Alfonso García-Pérez

There are errors in the in-text citations in the legend for Figure 1. Citations 1 and 14 should refer to 2 and 27, respectively. The correct Figure 1 legend is: A, Contrast in orientation between DPA [2] and SLA (blue arrows). Notice how two parallel specimens with identical longitudinal axes (SLA) show divergent DPA of 30°. B, Some examples from [27] showing MBRA (black arrows), DPA (blue lines) and SLA (green arrows). Notice the divergent angles.

